# Evidence for food-related and non-food-related maladaptive preference in a mouse model of binge eating disorder

**DOI:** 10.3389/fnbeh.2025.1653807

**Published:** 2025-10-20

**Authors:** Daniela Vajdová, Janet Ježková, Petra Procházková, Radka Roubalová, Enrico Patrono

**Affiliations:** ^1^Center for Advanced Behavioral Research (CABR), School of Psychology, University of New York in Prague (UNYP), Prague, Czechia; ^2^Institute of Microbiology of the Czech Academy of Sciences, Prague, Czechia; ^3^First Faculty of Medicine, Charles University, Prague, Czechia

**Keywords:** binge eating disorder, maladaptive decision-making/preference, environmental stressors, cortisol levels, palatable food

## Abstract

**Introduction:**

Rising numbers of binge eating disorder (BED) cases and excessive associated economic costs, together with the absence of efficient treatment strategies, highlight the importance of research in this area. To date, numerous studies have investigated the role of aberrant motivation in compulsive, maladaptive feeding behaviors. However, other aspects of maladaptive preference toward foods, possibly involving risk-based decision-making processes, are not yet fully elucidated.

**Methods:**

In this research, two types of environmental stressors—food-related and non-food-related—are explored in their ability to model compulsive behavior toward palatable food in mice.

**Results and discussion:**

Results from the behavioral experiments suggest that both types of stressors, when paired with the availability of highly palatable food, can produce aberrant motivation toward such food. These findings were subsequently supported by data obtained from cortisol concentration analysis in subjects.

## Introduction

Altered eating patterns and maladaptive feeding behaviors have been present in our society for some time. According to DSM-5, the central aspects of BED are episodes of binge eating occurring even when a person is not necessarily hungry, and cannot be stopped or postponed, with no compensatory behaviors, such as purging or diet and physical exercise (American Psychiatric Association, 2013). Several studies recognize exposure to stress as a contributing factor in the development of compulsive eating behavior (Volkow and Wise, 2005; Latagliata et al., 2010; Duarte et al., 2014; Patrono et al., 2015). For example, the consequences of food-related stressors, such as caloric restriction, are mediated by the effects of stress hormones, including cortisol and the corticotropin-releasing factor (CRF) (Dallman et al., 2003; Cottone et al., 2009; Iemolo et al., 2013; Micioni Di Bonaventura et al., 2014). Moreover, caloric restriction has been shown to model compulsive seeking and consumption of palatable food, driven by maladaptive motivation toward chocolate, rather than merely maintaining energy homeostasis (Latagliata et al., 2010; Patrono et al., 2015). Furthermore, in the past 20 years, several animal models of binge eating have been established where combinations of acute or chronic stress exposure were used (Hagan et al., 2003; Corwin and Buda-Levin, 2004; Bello et al., 2009; Brown and James, 2023), and human studies indicated that most individuals increase food intake during stress and that eating disorders usually emerge after a period of caloric restriction (Adam and Epel, 2007). In addition, over the years, a model of chronic subordination stress (CSS) has been developed by which subordinate animals develop a complex behavioral and metabolic syndrome associated with hyperphagia, vulnerability to obesity, metabolic-like, and type-2 diabetes-like syndromes (Bartolomucci et al., 2005, 2009; Razzoli et al., 2015). Therefore, it is possible that chronic, rather than acute, exposure to caloric restriction can better model a maladaptive salience state, where aberrant motivation and hedonic dysregulation may induce imbalanced eating behaviors.

On the other hand, non-food-related stressors, such as physical restraint, social defeat, subordination, or social isolation, have a crucial role in the release of adrenocorticotropic hormone (ACTH) and corticotropin-releasing hormone (CRH), which induce anxiety-like behaviors connected with maladaptive eating behaviors (Razzoli et al., 2015; Molina-Hidalgo et al., 2023; Ding et al., 2021; Cortés-García et al., 2022). Therefore, following exposures to various environmental stressors increases the release of stress hormones, even at lower exposure levels (Pankevich et al., 2010), suggesting that, whether food-related or non-food-related, stress may “reprogram” the brain’s salience network, leading to behavioral changes in food consumption (Jacques et al., 2019; Simon et al., 2024; Rehn et al., 2025). Interestingly, it has been found that exposure to stressful events affects decision-making toward more palatable, high-sugar foods (Ha and Lim, 2023). Decisions regarding food values, taste, and wellbeing conditions are encoded in the ventromedial prefrontal cortex (vmPFC), which is involved in computing reward value (Lim et al., 2023). Diminished self-controlled decisions in response to stress heighten sensitivity to food cues and reward values, leading to increased craving for palatable foods and disinhibited eating behavior in people with obesity as well (Jastreboff et al., 2013). However, whether food-related or non-food-related stress may induce altered, compulsive decision-making toward seeking and taking palatable food, such as milk chocolate, is yet to be fully elucidated.

In our study, using a well-established mouse model of compulsive chocolate-seeking and taking behavior, we aimed to investigate whether food-related and non-food-related stressors elicit similar behavioral patterns of compulsive chocolate-seeking and taking behaviors. Moreover, we hypothesize that food-related and non-food-related stressors elicit similar levels of cortisol release in the blood serum of stressed mice during the compulsive milk chocolate-seeking and taking behaviors.

## Materials and methods

### Animals

In our experiments, 48 adult male mice of the C57BL/6 strain were used (10 weeks old at the beginning of the experiment). According to the genetic conditions of the mice (inbred strain) and the reductionist approach mandated by the 3Rs (Replacement, Reduction, and Refinement), we determined that eight subjects per group were sufficient to achieve good levels of statistical validity, given the reduced variability in the behavioral assessments. Moreover, we performed a G*Power analysis (Version 3.1.9.7), which indicates that for an effect size greater than 0.59, the design has 95% power. Nonetheless, a higher number of subjects per group would further reduce the potential variability. Mice were obtained from the breeding colonies of the Institute of Microbiology of the Czech Academy of Sciences. All experiments were performed following procedures approved by the Institute of Microbiology Animal Care and Use Committee (approval ID 33-2023-P). At the beginning of the whole study, the mice were group-housed.

**Figure 1:**
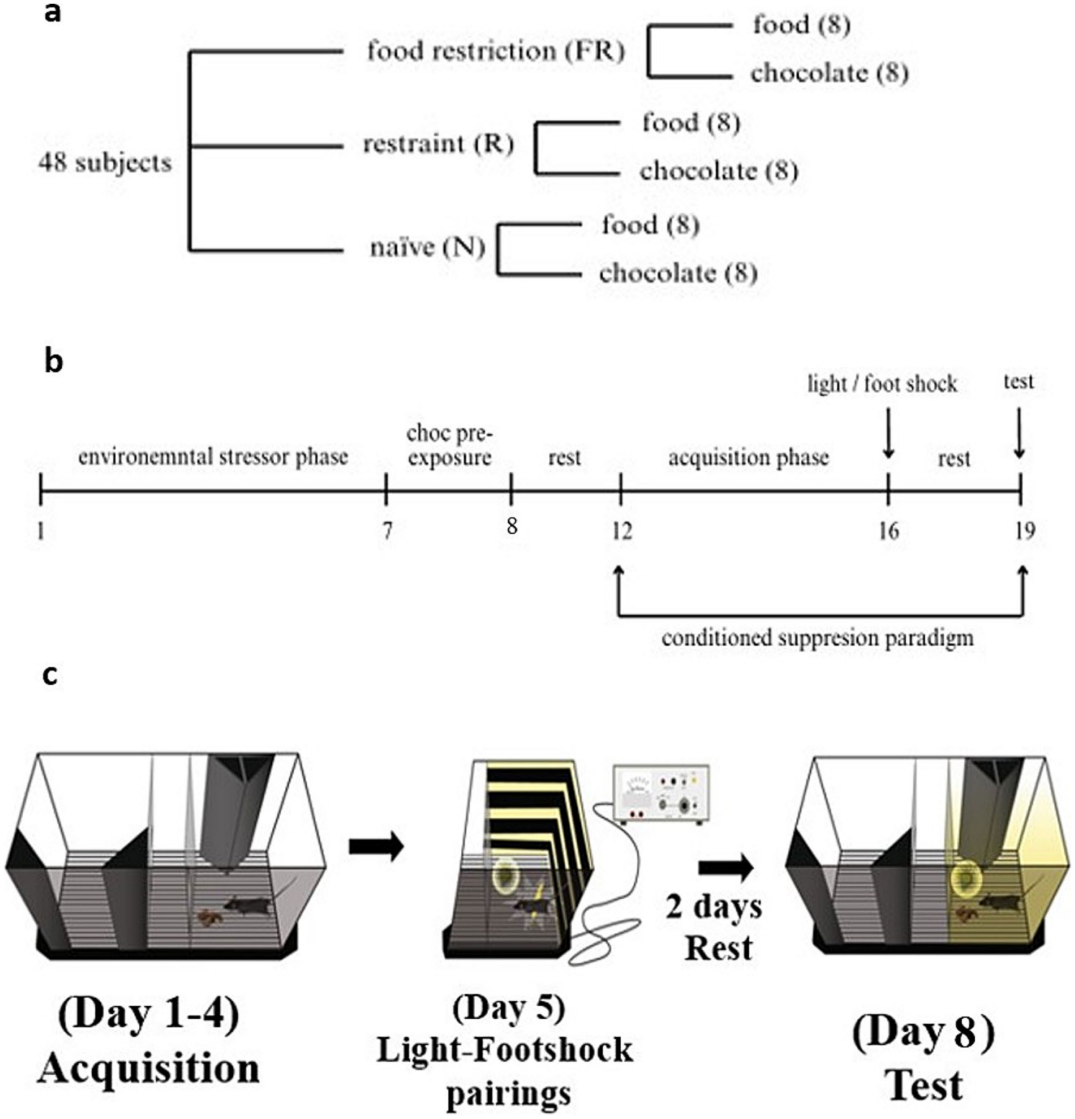
The groups, the experimental timeline, and the CSP protocol. **(a)** Groups of animals used in the experiments. Three main conditions were tested: the food-related stressor (food restriction, FR), the non-food-related stressor (restraint, R), and the control group (naive, N). For each condition, two groups (*n* = 8) were used: standard diet (food) and milk chocolate (choc). G*Power analysis showed an actual power of 0.95. **(b)** Timeline of the behavioral experiment in days. **(c)** The CSP apparatus and the protocol. A representation of the CSP apparatus with a description of the protocol used in this study (modified from Ventura et al., 2013).

The mice were divided into two groups, based on the environmental stressor condition: food restriction (FR) and restraint (R). A third group was used as a control group with no environmental stressor, naive (N). At the beginning of the caloric restriction protocol and throughout the entire procedure, FR mice were single-housed to control the feeding regimen, whereas R mice and N mice were group-housed. Each group was then divided into subgroups based on the stimuli provided: the standard diet (food) and the chocolate (choc) groups (Figure 1a). Because the mice were subjected to procedures concerning calorie intake and energy expenditure, they were weighed throughout the entire experimental protocol.

### Types of environmental stressors

A mild, chronic caloric restriction is a standard paradigm for modeling compulsivity toward palatable food in animals, especially rodents (Volkow and Wise, 2005; Adam and Epel, 2007; Latagliata et al., 2010; Duarte et al., 2014; Patrono et al., 2015). Here, caloric restriction was terminated 13 days prior to the test day. Thus, at the time of the test, the mice were satiated and not considered to have energy homeostasis needs. On the other hand, physical restraint in mice is a commonly used experimental stressor in research. In the current study, we aim to investigate whether a non-food-related stressor, such as restraint, influences the seeking and consumption of palatable foods. To maximize the comparison of chronicity between the food-related stressor (caloric restriction) and the non-food-related stressor (restraint), we considered using the same duration (7 days) for both stressors.

### Timeline of the behavioral experiments

Experimental procedures were divided into three distinct phases (Figure 1b):

1. Environmental stressor phase (days 1–7): Groups FR and R, from both the choc and food groups, were exposed to environmental stressors, while the two *N* groups (choc and food) remained undisturbed in their cages.
2. Brief exposure to the chocolate (day 8–9): All groups of mice received *ad libitum* access to milk chocolate in their home cages to reduce the possibility of novelty effects toward chocolate (Patrono et al., 2015).
3. Rest days (10–11): mice were left undisturbed in their home cages. FR mice were kept in single-housed conditions, while the R and the N mice were group-housed.
4. Conditioned suppression paradigm phase (days 12–19): All groups of mice underwent the same behavioral procedure. The food groups were exposed to 1 g of standard diet (Ssniff; Spezialdiäten GmbH, Germany), and the choc groups were exposed to 1 g of milk chocolate (Milka, Mondelēz International, Switzerland). This phase ended with a test on the final day (19). All the mice were sacrificed at the end of the test, and blood samples were collected for further biological analysis.

### Environmental stressor phase

On day 1 of the experiment, the environmental stressor started for the FR and R groups (days 1–7). Throughout the week, mice from the FR group were single-housed and exposed to a mild-chronic food restriction, consisting of a caloric-restricted intake designed to lose ~15% of their starting weight, once a day (11 a.m.). After 7 days, animals from the FR group were given *ad libitum* access to standard food, regaining their previous weight within 24 h.

Animals from the R group were exposed to a mild, chronic, non-food-related restraint protocol. In this protocol, mice were placed in tubes (50 mL Falcon tubes, appropriately modified with holes allowing airflow and tail retention) for 1 h daily in dark and sound-attenuated conditions. In this study, physical restraint was used to simulate the psychological and physiological effects of seclusion-induced stress, which is reported to lead to behaviors like anxiety and depression (Han et al., 2018; Ding et al., 2021). The R group was given *ad libitum* access to a standard diet and was kept in group housing.

### Conditioned suppression paradigm

The phase of behavioral experiments consisted of the exposition to a conditioned suppression paradigm (CSP) (days 12–19), which is a previously established and validated method for producing and testing compulsive behavior toward palatable food in mice (Latagliata et al., 2010; Patrono et al., 2015; Di Segni et al., 2014) (Figure 1c). The whole task was performed in a sound and light-attenuated room. The CSP consists of four distinct conditions: acquisition phase (4 days), light-footshock conditioning paradigm (1 day), rest (2 days), and test (1 day). On each acquisition day, food was removed from the cages 2–3 h before the behavioral procedure to prevent satiation throughout the task, without inducing starvation (Carper et al., 2020). The apparatus for the CSP was formed of two gray Plexiglas chambers (15 cm × 15 cm × 20 cm) and a central alley (15 cm × 5 cm × 20 cm) with a stainless-steel grid floor. In each chamber, two triangular parallelepipeds (5 cm × 5 cm × 20 cm) were arranged in different patterns (always covering the same surface of the chamber) and placed to facilitate the animals’ ability to distinguish between the two chambers (Latagliata et al., 2010; Patrono et al., 2015). The apparatus lay on a grid floor with LED stripes, which were later used to provide the light-footshock pairings. During the acquisition phase, all animals were placed individually into the apparatus, and 1 g of food or chocolate was presented on a cup (3.8 cm) in one of the rooms (the reward room). An empty cup was also placed in the non-rewarded chamber. The visual pattern enabled the mouse to associate the chamber with the presence of food or chocolate. For the following 4 days, mice were placed in the central alley, with the sliding doors open, and they spent 20 min daily in this apparatus while being recorded by a camera. The time spent in each compartment and the number of entrance occurrences were measured.

On the 5th day of CSP, there were light-footshock pairings (Latagliata et al., 2010; Patrono et al., 2015). All the mice were individually placed and isolated in each chamber of the apparatus, which was equipped with different visual patterns compared to those used during the acquisition phase. The light (50 lux) served as the conditioned stimulus (CS), and the footshock as the unconditioned stimulus (US). The light-footshock pairings protocol was as follows: over a total time of 30 min, the first 10 min, mice were exposed every minute to the following 1-min conditioning protocol (40 s, light off; 20 s, light on; 1 s, 0.4 mA scramble footshock). Following this, the mice remained confined in the apparatus for 10 min, followed by another 10 min of the previous light-footshock protocol. After this, animals were returned to their home cages and given 2 days of rest.

On day 8 of the CSP, a test day was conducted during which the risk-based decision-making process of seeking and consuming chocolate or food despite the presence of light, which signaled an adverse event, was assessed (Latagliata et al., 2010; Patrono et al., 2015). Animals were food-deprived for 2–3 h, as in the acquisition phase. At the start of the task, mice were placed in the alley. The reward was placed in the same chamber used during the acquisition phase, and the LED light (CS) was turned on in the baited chamber, following the same schedule as the previous light-footshock pairing but without the footshock (US), and a 10-min rest, for a total of 20 min. Again, the time spent in each chamber and the occurrence of entrances were recorded and used for behavioral analysis, as well as the consumption of rewards. At the end of the task, mice returned to their home cages, and after 60 min, blood samples were collected.

### Cortisol concentration analysis

Serum from sacrificed animals was used to determine cortisol concentrations immediately after the final test in the FR and R groups. Our goal was to evaluate whether specific exposure to the CS, coupled with food or chocolate, would have an acute effect on stress hormone release in mice that were previously sensitized to stressful conditions. For this reason, we decided to measure cortisol levels only in the FR and the R groups that were exposed to food or choc. However, in order to estimate a basal measure of cortisol release in mice used in these experiments, serum samples were collected from mice (*N* = 6) of the same litters that were not exposed to any condition (control cage, CC). Although corticosterone is considered the primary glucocorticoid involved in regulating stress responses in rodents, researchers often opt to detect cortisol as a stress indicator. Several studies have observed increased cortisol levels in mice following stress, using cortisol as an index of stress activation (Gong et al., 2015). For this procedure, the commercial Mouse Cortisol ELISA Kit was used, following the manufacturer’s suggested protocol. The serum samples were used undiluted for the assay. Cortisol was measured in picograms per milliliter of serum (pg/mL).^1^

### Data analysis

Video recordings obtained from acquisition days and test days were analyzed using EthoVision XT 17 software (Noldus), which is used for tracking animal behavior. Data derived from this software were subsequently analyzed using R Studio for fundamental analysis and assumption tests, and GraphPad Prism 9.1 software for more complex statistical analyses. Two-way ANOVA with repeated measures (RM) was performed to determine any significant differences among groups (FR, R, and N) and also within different compartments of the apparatus (reward, alley, and no reward) in terms of total time spent, measured in seconds, as well as the number of occurrences (how many times the animal entered specific room). Finally, a one-way ANOVA was performed to determine if there were any significant differences in consumption between groups, measured in grams. Additionally, a one-way ANOVA was conducted to assess differences in cortisol levels between the FR and R groups.

## Results

### Behavioral experiments

**Figure 2:**
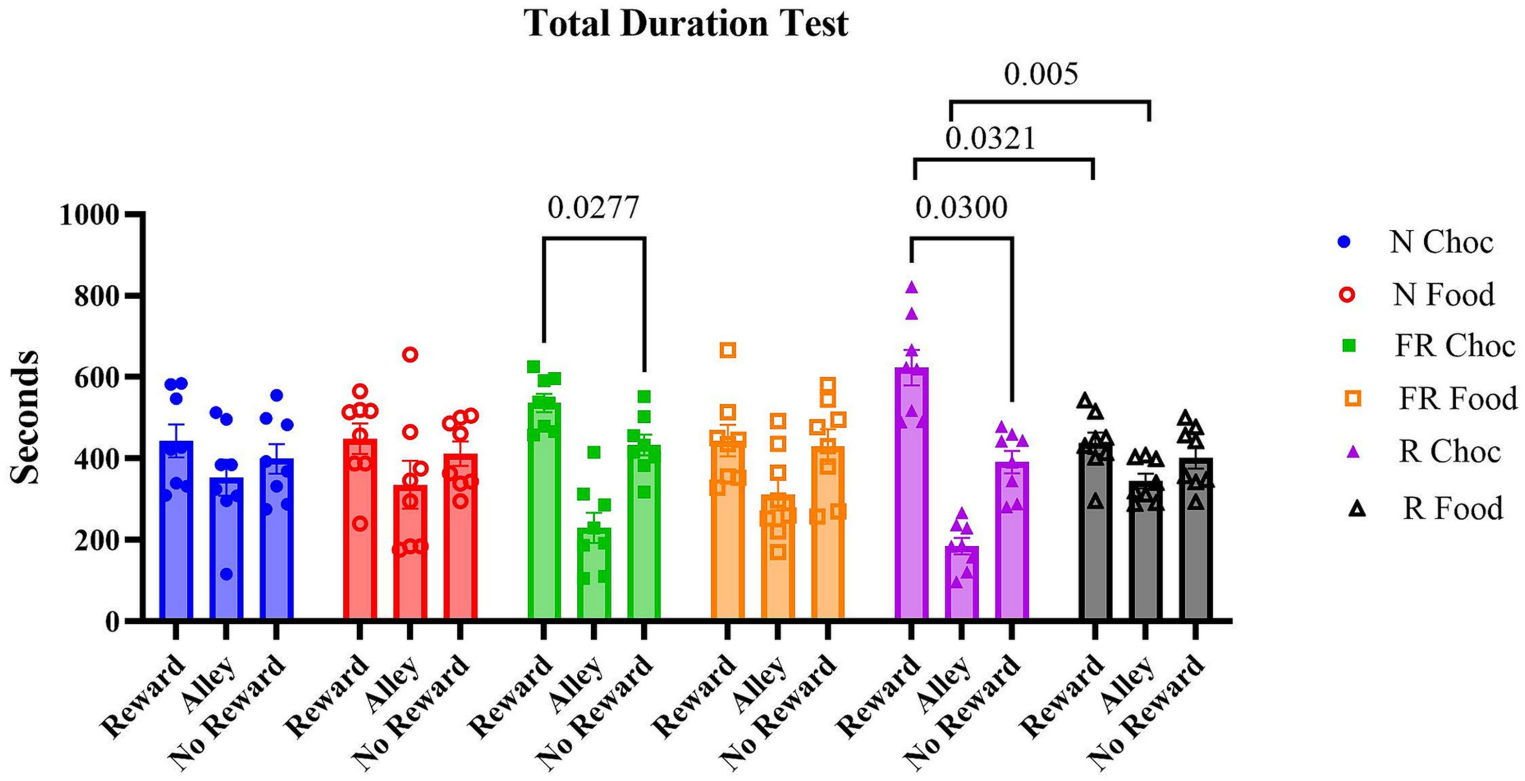
The time spent in the apparatus during the test day. The graph represents the time duration in each compartment for all groups in the experiment. Error bars represent ± SEM.

Firstly, the time spent in each compartment was measured during the test day. A two-way ANOVA revealed that, overall, the time spent in each compartment was significantly different [F (1.887, 79.26) = 30.59; *p* < 0.0001], but with no significant difference among the groups [F (5, 42) = 1.237; *p* = 0.3091], and with a significant compartments X groups interaction [F (10, 84) = 2.856; *p* = 0.0042], with multiple comparisons showing that only the FR Choc (*p* = 0.0277) and R Choc (*p* = 0.03) groups showed a significant preference for the chocolate-paired compartment during the test (Figure 2). These results suggest that prior exposure to environmental stressors, whether food-related or non-food-related, and the subsequent administration of palatable food, compared to non-palatable food, induced a maladaptive, risk-based decision to spend more time in the reward room, despite the presence of a conditioned stimulus signaling the adverse consequences. Conversely, the control groups, whether exposed to the stressors or not, or whether they received chocolate or standard food, did not exhibit any signs of compulsive seeking or taking behavior, suggesting that neither the stressor nor the chocolate alone is capable of inducing a maladaptive, compulsive reward-based decision-making behavior.

**Figure 3:**
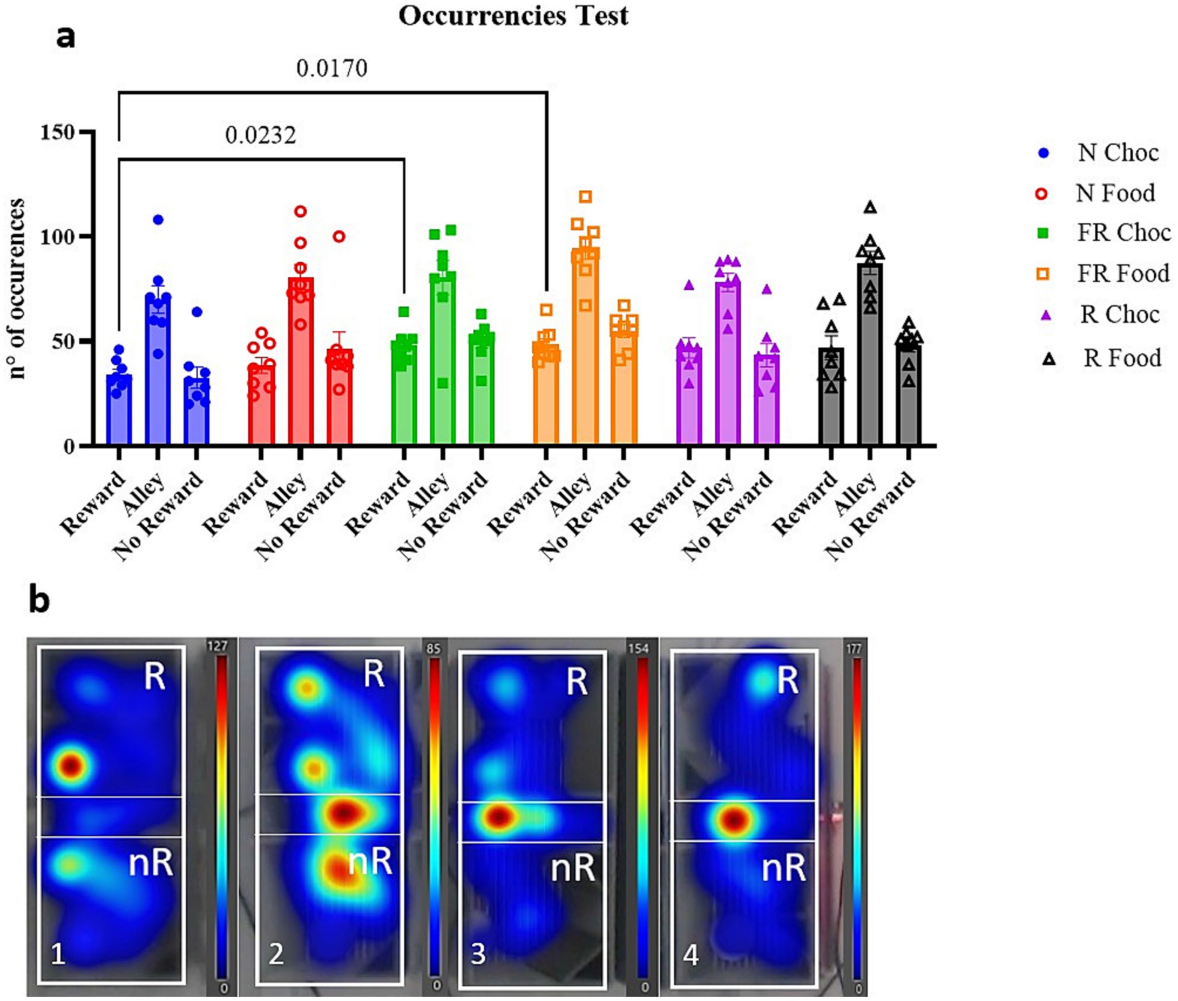
The occurrences in the three compartments during the test day. **(a)** Represents the graph with the number of entries in each compartment for all groups in the experiment. Error bars represent ± SEM. **(b)** Shows heatmap plots of four representative examples of the locomotory activity of the mice in the apparatus throughout the 20-min session. (1 = FR Choc; 2 = R Choc; 3 = N Food; 4 = N Choc). The bars represent the number of occurrences.

Following, the number of times (occurrences) in which the mice entered the three compartments was measured to evaluate whether the previous data regarding the time spent in the three compartments was biased by the presence of the negative CS (light). A two-way ANOVA revealed noticeable significant differences among the chambers [F (2, 21) = 90.69; *p* < 0.0001] because of more passages throughout the alley (Figure 3a, not shown), and a significant difference between the groups [F (3.435, 72.14) = 5.617; *p* = 0.0010], with further multiple comparisons showing that the reward compartment was accessed significantly more times by the FR Choc group (*p* = 0.0232) and the FR Food group (*p* = 0.0170) than N Choc group (Figure 3a). In Figure 3b, representative heatmaps of occurrences throughout the entire apparatus for the entire 20-min session are shown (Figure 3b).

**Figure 4:**
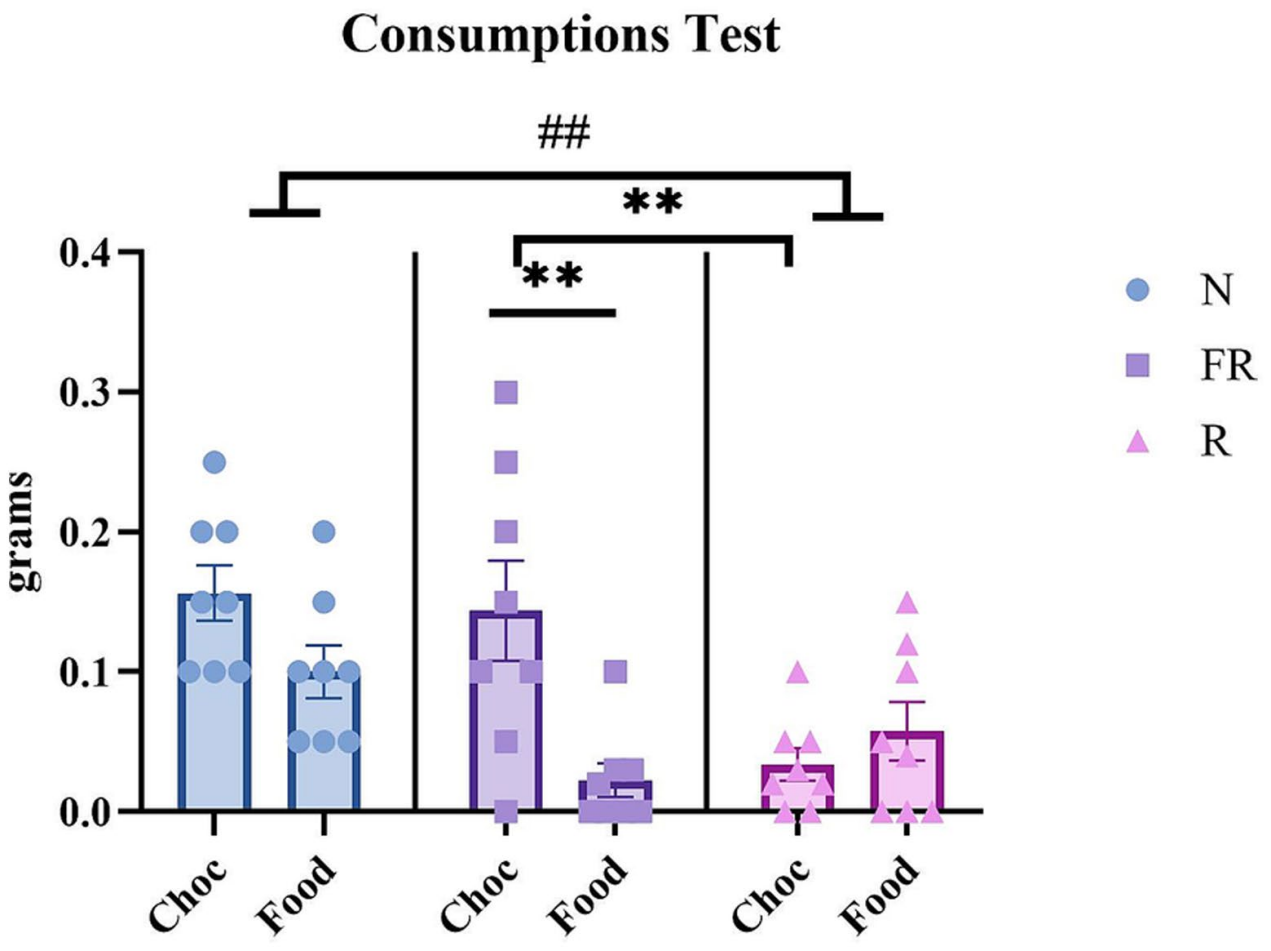
The amount of food/chocolate consumed during the test day. The graph represents the consumption of food or chocolate in all the groups of mice during the test. A difference between the N and R groups (## = *p* > 0.01). Moreover, the FR Choc group showed a significant difference in consuming chocolate with respect to the FR Food group (** = *p* > 0.01) and the R Choc group (** = *p* > 0.01). Error bars represent ± SEM.

Finally, the amount of food and choc consumed during the test was recorded and measured. A two-way ANOVA revealed significant differences in consumption between the groups [F (1.740, 13.92) = 4.683; *p* = 0.0317], but not among the rewards (food or choc) [F (7, 8) = 0.2084; *p* = 0.9736] or their interaction [F (14, 16) = 0.6258; *p* = 0.8081]. Multiple comparisons revealed a significant difference between the N and R groups (*p* = 0.0026) (Figure 4). Further, a one-way ANOVA was used to compare the consumption of standard food and chocolate within the groups, revealing that the FR Choc group consumed significantly more chocolate than the R Choc group (*p* = 0.0046) and the FR Food group (*p* = 0.0015).

### Cortisol levels

**Figure 5:**
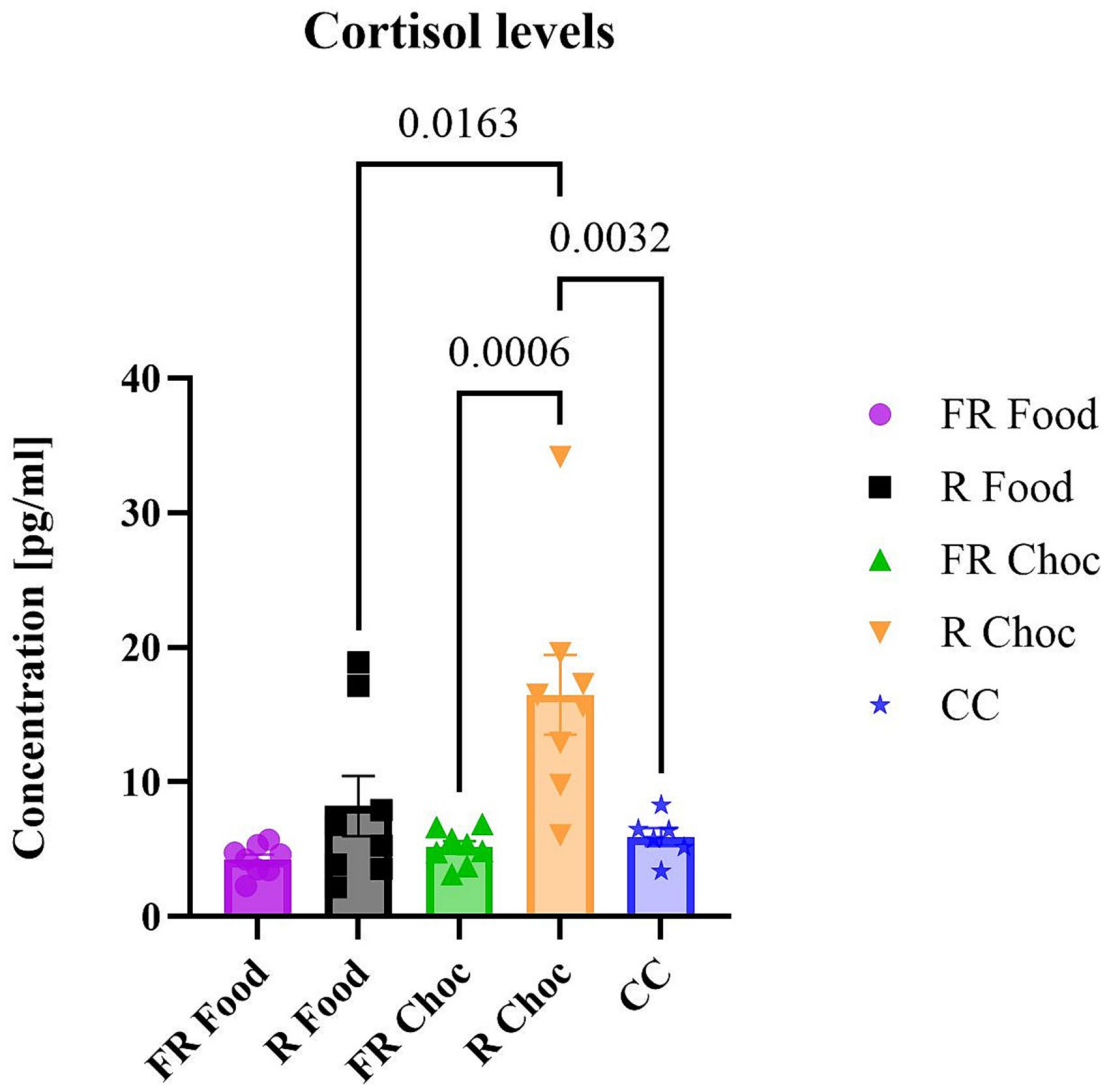
The levels of cortisol. The graph represents the concentration of cortisol in the serum of the groups exposed to the stressors. Error bars represent ± SEM.

In this study, we aimed to evaluate whether specific exposure to light (indicating adverse footshock) coupled with food or chocolate would have an acute effect on stress hormone release, such as cortisol, in mice previously sensitized to stressful conditions. For this reason, we decided to measure cortisol levels only in the FR and the R groups that were exposed to food or choc. However, in order to estimate a basal measure of cortisol release in mice used in these experiments, serum samples were collected from mice (*N* = 6) of the same litters that were not exposed to any condition (control cage, CC). The one-way ANOVA revealed significant differences between the FR and R groups paired with chocolate [F (4, 33) = 7.986; *p* = 0.0001] (Figure 5). In Tukey’s multiple comparisons *post hoc* test of cortisol concentration, animals from the R Choc group had significantly higher cortisol concentrations (*p* = 0.0006) compared to those from the FR Choc group, R Food group (*p* = 0.0163), and the CC group (*p* = 0.0032). These results show that, despite low chocolate consumption, the non-food-related group (R) exhibited higher levels of stress compared to the food-related group.

## Discussion

The primary objective of this study was to investigate whether food-related or non-food-related stressors could influence risk-based chocolate-seeking and taking behavior, which can be regarded as a form of maladaptive preference toward palatable foods. When choosing the experimental, palatable food to face the standard chow, we decided to feed the mice milk chocolate because, as stated elsewhere (Latagliata et al., 2010), it is considered a mild, palatable food with a complex combination of fat and sugar, somewhat energy-based rather than high-sucrose or high-fat food. That, to our conditions, best represents ecologically the human preference for palatable food. To this end, the study employed an established paradigm, the CSP (Latagliata et al., 2010; Patrono et al., 2015). The use of food-related and non-food-related stressors affected the maladaptive risk-based chocolate seeking/taking behavior differently during the testing condition, where an aversive CS (light) was paired with the chocolate. Under these conditions, the mice chose to explore and spend more time in the chocolate-paired compartment, despite the presence of the light. It appeared that both stress groups (FR and R) paired with chocolate developed risk-based compulsive chocolate-seeking behavior (Figure 2), a sign of maladaptive preference toward palatable foods (Latagliata et al., 2010). A large body of evidence has demonstrated that dopamine, among other neurotransmitters, plays a crucial role in modulating the hedonic value of both natural and pharmacological rewards (for review, Volkow et al., 2011). The dopamine release in limbic structures such as the ventral tegmental area and the nucleus accumbens toward more cortical areas such as the orbitofrontal cortex and the anterior cingulate cortex is responsible for the self-reported level of pleasure derived from eating the food (Small et al., 2003; Norgren et al., 2006; Epstein et al., 2009; Schultz, 2010). A recent study hypothesized that low accumbal dopamine D2 receptor availability is a “constitutive” genetic risk factor for compulsion-like eating behavior, representing a potential neuroadaptive response that parallels the shift from motivated to compulsive eating (Patrono et al., 2015). Thus, it is possible that in the current study, the shift toward a maladaptive seeking for chocolate in both food-related and non-food-related stressed mice induced altered expressions of dopaminergic activities in the mesocorticolimbic circuit of motivation.

Moreover, significant differences in consumption were observed between the “chocolate groups” (Figure 4), suggesting that the distinct nature of the stressors distinctly influenced consummatory preference for palatable food. Mice subjected to FR were highly driven by the palatability of the stimulus and consumed significantly more chocolate (consummatory behavior, “liking”). Here, it is crucial to note that the FR mice were single-housed throughout the procedure and the CSP. In this study, therefore, the use of a single-housed FR complicated the interpretation of the results due to the social isolation aspects involved in the FR protocol with respect to the effects of the non-food-related physical restraint stressor. The two procedures differ not only in their relationship to food, but one of them also involves social isolation, and therefore, it is difficult to interpret whether the maladaptive chocolate consumption is caused by an emotional stressor, such as being housed alone in a cage, or by a more physical stressor, such as having experienced hunger or being restrained in a tube. Thus, the comparison between the FR and the R groups must be carefully evaluated, solely from the perspective of maladaptive chocolate-seeking behavior. The results of this study propose that maladaptive chocolate-seeking behavior (appetitive behavior) may not be induced solely by food-related stressors, but also by other types of stressors. On the other hand, the maladaptive chocolate-taking behavior (consummatory behavior) cannot be fully explained by the two stress procedures presented here, due to the lack of control in the social isolation aspects seen in the FR protocol, compared to the R protocol. In normal conditions, appetitive and consummatory behaviors are expressed together in a balanced proportion. However, in pathological conditions, such as drug addiction, the expression of “wanting” (appetitive) and “liking” (consummatory) is imbalanced (Robinson and Berridge, 2003). Recently, it was discussed whether dissociations between “wanting” and “liking” could be tested, and it was shown that, although animal research has shown it is possible to want a reward that is not liked, human research has produced contradictory results, suggesting that “expected pleasantness” (cognitive desires based on predictions of future pleasure) represents a source of confound for wanting and liking operationalizations in humans (Pool et al., 2016). Expected pleasantness does not correspond to animal liking, a hedonic experience, or to animal wanting, which relies on affective relevance, consisting of the perception of a cue associated with a relevant reward for the organism’s current physiological state. In the current study, we considered that although wanting and liking are often expressed together, exposure to different environmental stressors may have imbalanced their expression, leading to maladaptive appetitive behavior. Nevertheless, as previously described, the same exposure to different environmental stressors cannot explain the possibly altered consummatory behavior seen in the FR group, due to a lack of control in the social isolation condition.

On the other hand, the behavior of mice in the R group was less driven by the “liking” of the chocolate and more driven by the motivation to reach the chocolate (appetitive behavior, “wanting”), where the palatability of the stimulus played a lesser role for them. Generally, protocols of physical restraint in rodents are used to study behavioral and physiological effects of stress associated with forced isolation (seclusion) (Ding et al., 2021). Physical restraint and forced isolation can cause stress-induced behavioral and physiological changes, mirroring some of the effects seen in voluntary social withdrawal, possibly producing similar behavioral responses and coping strategies, particularly those related to depression-like and anxiety-like behaviors, such as immobility and aversion (Ribeiro Do Couto et al., 2006; Lee et al., 2021). Nonetheless, although physical restraint can produce central and peripheral responses likely shared with many other “social” stressors (such as forced swimming, foot-shocks, air puff, or social defeat), it is known that physical stress differently affects reward behaviors compared to emotional stress (Ramsey and Van Ree, 1993; Pijlman et al., 2002; Schettino et al., 2024). Moreover, it has been stated elsewhere that it is not possible to make general claims about the effect of social stress or physical stress on food intake because stressors of the same class can have different effects on food intake in rodents (François et al., 2022). Nevertheless, the findings of this study suggest that there are differences in how various types of environmental stressors, including food-related and non-food-related stressors, modulate maladaptive preference toward palatable food. Moreover, it is interesting to note the difference in consummatory behavior induced by a food-related stressor compared to a non-food-related stressor. Food-related stressors may engage the compulsivity system differently than non-food-related stressors, with “compulsive taking” being considered a consummatory behavior (liking) and “compulsive seeking” being considered an appetitive behavior (wanting) (Lenoir et al., 2007; Latagliata et al., 2010; Boggiano et al., 2013; Patrono et al., 2015; Brown and James, 2023). As previously discussed, the maladaptive eating behavior shown here seems to be independent of energy homeostatic needs because the stressful conditions (FR and R) were terminated 13 days before the test day, and because the prior-to-the-session fasting (2 h) was unable to induce any homeostatic change. Therefore, the complex “compulsive taking” and “compulsive seeking” appear to contribute to encoding anticipated outcomes and thus may influence future risk-based decisions. Therefore, it is possible to argue that exposure to food-related stressors might be a marker for compulsive, risk-based behavior, which can be associated with maladaptive decision-making. The analysis of the occurrences in each chamber (Figure 3a) throughout the whole 20-min test session reveals no differences in exploration among the three chambers, particularly between the reward and non-reward chambers. Data analysis of the time spent in each compartment during the test in the “light windows” revealed the same pattern as the analysis of the entire 20-min session (data not shown), suggesting that the mice did not avoid the light and entered the reward room despite its presence. To better visualize this, we plotted heatmaps of four representative mice, with the red areas indicating the most frequently visited regions (Figure 3b). Thus, considering the results from the time spent in each compartment during the test, it is suggested that all the groups explored the chambers equally during the test session (except for the alley, which showed more occurrences because it is in the middle of the apparatus). However, the FR Choc and the R Choc spent more time in the reward chambers compared to the non-reward chambers, further suggesting that, despite the precise scheduling of the light, potentially signaling the negative consequence of a footshock, the stressed mice significantly preferred to seek chocolate, and that this preference was not related to the light schedule associated with the adverse event. Connecting these findings, it can be argued that aberrant motivation and appetitive behavior (the “wanting”) were indeed developed in both FR and R groups associated with chocolate. However, when evaluated from the perspective of maladaptive decision-making, model-free preference, only the FR group of mice showed maladaptive consummatory behavior, as a shift in behavior from “wanting” to “liking” was evident (Jastreboff et al., 2013; Goschke, 2014; Patrono et al., 2016; Ha and Lim, 2023). Their behavior became more consummatory than just appetitive, showing a strong preference for the higher palatability of chocolate. Notably, the FR and N groups do not differ significantly in their chocolate consumption. However, it is also observed that the standard food consumption between the FR and N groups differs, although the difference is not statistically significant. Moreover, examining the data of the R group, consumption is significantly lower in both chocolate and food. This can be explained by the fact that milk chocolate is independent of other factors. At the same time, the seeking and taking behavior despite a dangerous signal (light) is specific to prior exposure to environmental stressors. Furthermore, as it was previously established, a mild caloric restriction is a mild food-related stressful event that affects the motivation toward palatable foods, transforming a usual preference for palatable foods into a maladaptive, compulsive binge eating behavior, aligning with the literature that highlight the role of a food-related stressful event in impinging on maladaptive preference toward palatable foods (Lenoir et al., 2007; Latagliata et al., 2010; Boggiano et al., 2013; Patrono et al., 2015; Brown and James, 2023; Jacques et al., 2019; Ha and Lim, 2023; Lim et al., 2023).

Furthermore, it was demonstrated here that the R group of mice associated with chocolate exhibited a significant preference for palatable food, despite the light signaling the foot-shock, which, however, was not accompanied by consummatory behavior. Therefore, it is possible that forms of “physical restrictions” have a role in eating disorders, especially those involving compulsive taking of palatable, comfortable food. Regarding the possibility that physical restrictions mimicking forced isolation might produce similar stress-induced behavioral and physiological effects, a pattern of bidirectional associations between social withdrawal and disordered eating has been found, varying by time points, gender, and type of eating problem (Cortés-García et al., 2022; Possa-Paranhos et al., 2023; Mason, 2024). Moreover, the results of cortisol concentration showed that the R group paired with chocolate had significantly higher serum cortisol levels compared to the FR group, which received the same stimulus. A substantial body of evidence links the hormone cortisol to the modulation of feeding behaviors in both animals and humans (Adam and Epel, 2007). In these studies, the indulgence in palatable food was viewed as a way to “self-medicate” and alleviate a negative emotional state that stressed individuals may experience (Dallman et al., 2003; Adam and Epel, 2007). Taking into consideration these notions, the cortisol concentration in animals from the FR Choc and Food, and CC compared to the R Choc group may be explained, as it is connected with the previously described higher consumption of chocolate, which is a form of indulging in palatable food to alleviate the negative state arising from the stressful environment (stress procedure). However, the consummatory behavior was not observed in the R Choc group, as their consumption was significantly lower and cortisol levels were significantly higher than in the FR Choc, Food, and CC groups. It has been shown that different lengths of exposure to physical restraint can alter serotonergic and cortisol levels in rodents (Price and Gorzalka, 2002) and that complex combinations of chronic vs. acute exposure to stress and intermittent access to palatable food may induce changes in blood glucose homeostasis (Buesing et al., 2025). Thus, it is possible that the higher levels of cortisol seen in the R Choc group (which was collected after the end of the test session) were induced by the presence of the aversive CS (light) that, as in the case of the FR Choc group, was not only considered as a CS for the footshock, but also as a “cue-stress trigger” from the previous physical restraint. It is important to note that both the stressed groups were exposed to the test sessions 13 days after completing the stress protocols.

Alternatively, since cortisol levels are indicative of the response to the aversive CS (light), the CS is effectively aversive only for the R Choc group. Thus, while the mice that underwent FR (in social isolation) are attracted to chocolate despite the presence of the aversive CS, because the food-related stressor eventually increased the hedonic value of the food (consummatory phase), the mice that underwent R are attracted only by the motivational value of the environment associated with food but not by the food itself (appetitive phase). This may indicate a dissociation between incentive salience attribution or “wanting” (a Pavlovian process) from the hedonic value attribution to the unconditioned stimulus or “liking.” As the two processes are typically coupled, their dissociation may be more indicative of maladaptive behavior (Berridge and Robinson, 2016). Finally, we demonstrated how different types of environmental stressors can modulate the stress response to palatable food (Chotiwat and Harris, 2006; Cacioppo et al., 2015). When related to humans, these findings highlight how environmental stressors of different natures can affect maladaptive compulsive seeking and taking behavior toward palatable foods in distinct ways. Particularly, how caloric restriction, compared to physical restriction, may impact future dietary choices. Also, these findings can add to some understanding of the issue of high failure rate in long-term weight management, as a history of caloric restriction may increase salience/decision-making for palatable food, but also provides some explanations for why negative emotional states and cognitions are common triggers for binge eating episodes in human patients with eating disorders.

### Limitations of the study

Despite these efforts, the research has some limitations. Firstly, a limitation is evident in the selected sample, which consists only of male mice. While the use of male mice continues to provide valuable data, according to Karp and Reavey (2019), incorporating female samples into research could enhance the translatability of findings from animal models to humans. Nonetheless, as previously discussed, it is well established that binge eating is 2–4 times more prevalent in females than in males (Klump et al., 2013; Carlin et al., 2016). Connecting these findings to the study of compulsive behavior toward palatable food, it would be interesting to explore this phenomenon in female mice as well (Klump et al., 2013; Anversa et al., 2020; Keski-Rahkonen, 2021).

Secondly, the use of physical restraint as a non-food-related stressor yielded results that did not fully clarify the causative relationship between stress and a maladaptive preference for palatable foods. Thus, in future works, a group exposed only to social isolation should be included.

Thirdly, this study aimed to evaluate stress-induced binge eating from the perspective of maladaptive, risk-based decision-making. However, the behavioral results showed that maladaptive seeking and taking of chocolate in mice is a form of maladaptive preference for palatable foods. That decision-making alone cannot explain pathological palatable food-seeking/taking behaviors, at least in animal models. Thus, it is essential to consider additional models that can distinguish between instances of preference and decision-making.

## Conclusion

In this study, we demonstrated that environmental stressors of different natures, particularly food-related and non-food-related, may elicit distinct responses toward the maladaptive seeking and taking of palatable foods, which is a primary hallmark of BED. Although this study revealed critical insights into distinguishing between different stressors that trigger maladaptive eating behaviors, it is far from being considered complete. For example, brain neurotransmission in the mesocorticolimbic system, including dopaminergic, serotonergic, and GABAergic activities, should be investigated. Moreover, further non-food-related stressors should be considered, such as social defeat or maternal separation. In conclusion, this study may serve as a procedural starting point for future research to develop more effective therapeutic tools for addressing maladaptive eating behaviors.

## Data Availability

The original contributions presented in the study are included in the article/supplementary material, further inquiries can be directed to the corresponding author.

## Footnotes

^1^ https://www.antibodies.com/catalog/elisa-kits/mouse-cortisol-elisa-kit-a87503
